# Supplementary figures and images for: Deletion of the Candida albicans TLO gene family results in alterations in membrane sterol composition and fluconazole tolerance
Source: PLoS One. 2024 Aug 9;19(8):e0308665. doi: 10.1371/journal.pone.0308665 (PMC11315338; doi:10.1371/journal.pone.0308665)

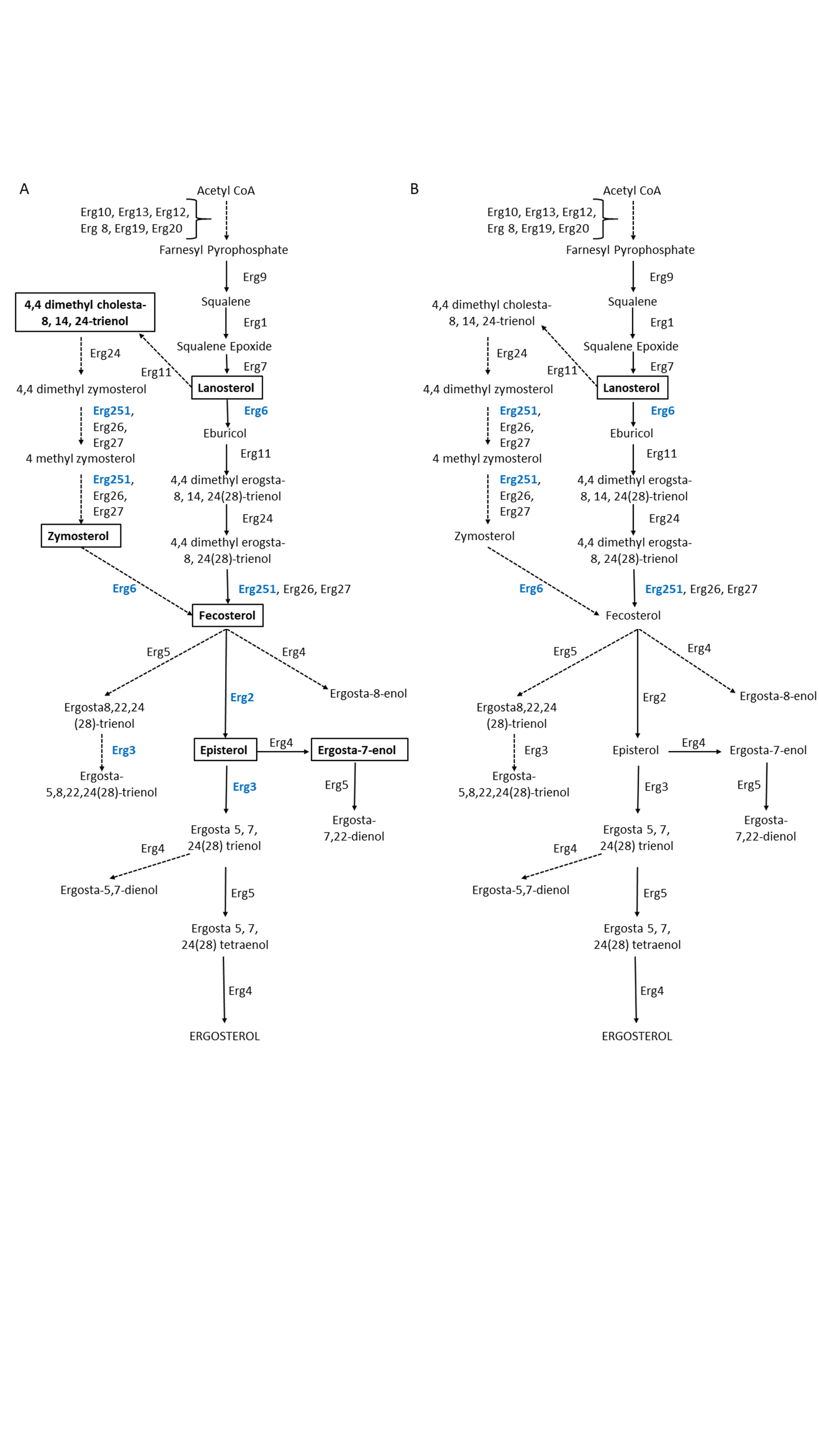

Supplement: S1 Fig — Sterol intermediates in bold signify their accumulation, the Erg proteins highlighted in blue signify the downregulation of the encoding ERG gene in the Δtlo mutant relative to the WT strain. In the absence of the drug, several toxic intermediates of the ergosterol biosynthesis pathway accumulated in the Δtlo mutant, such as lanosterol, 4,4-dimethyl cholesta-8, 14, 24-trienol, zymosterol, fecosterol and episterol. The accumulation of these intermediates coincides with the down regulation of the ERG genes encoding enzymes that catalyses their conversion, which are highlighted in blue (A). In the presence of the fluconazole, the build-up of these toxic sterol intermediates in the Δtlo mutant is alleviated, although differential ERG6 expression contributes to the build-up of lanosterol in the Δtlo mutant (B). (TIF) [file pone.0308665.s001.tif]

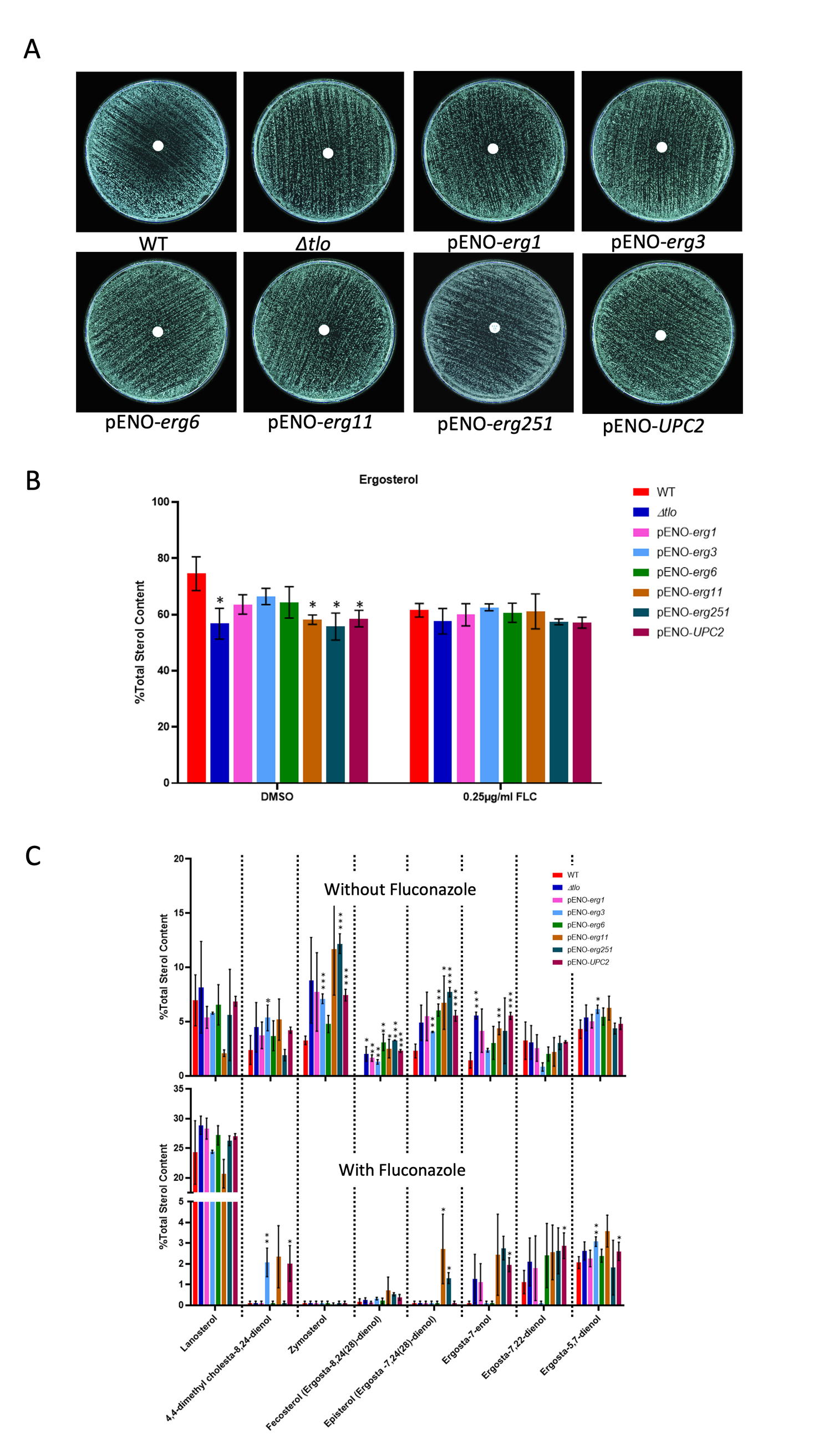

Supplement: S2 Fig — The sensitivity of several ergosterol gene overexpression mutants in the Δtlo mutant background was tested using fluconazole disk diffusion assays (A). Ergosterol levels of the WT strain, Δtlo mutant and ERG gene overexpression mutants in the presence and absence of fluconazole (B). Sterol composition of the WT strain, the Δtlo mutant and the ERG gene overexpression mutants in the absence and the presence of fluconazole (C). Asterisks indicate statistical significance; where * = p-value <0.05, ** = p-value <0.01 and *** = p-value <0.001 between the WT strain and the mutants tested. (TIF) [file pone.0308665.s002.tif]
