# Supplementary material for: Deletion of the Candida albicans TLO gene family results in alterations in membrane sterol composition and fluconazole tolerance
Source: PLoS One. 2024 Aug 9;19(8):e0308665. doi: 10.1371/journal.pone.0308665 (PMC11315338; doi:10.1371/journal.pone.0308665)
Supplement: Attachment — Submitted filename: Response to Reviewers.docx [file pone.0308665.s007.docx]

Division of Oral Biosciences

School of Dental Sciences &

Dublin Dental University Hospital

Trinity College

Dublin 2

Ireland

26^th^ July 2024

**RE: Resubmission of research article: PONE-D-24-27239R1**

**O’Connor-Moneley *et al.,* Deletion of the *Candida albicans TLO* gene family results in alterations in membrane sterol composition and fluconazole tolerance**

Dear Dr. Upadhya

We are very grateful for the rapid and thorough review of our manuscript. We are also grateful to the two reviewers for their very helpful comments and suggestions. We are particularly glad that they both agree that the manuscript describes a valuable contribution to our understanding of the role of the *C. albicans* *TLO* gene family in fluconazole tolerance and that it is suitable for publication. We have responded to and addressed all of the editorial and reviewers’ comments below and we hope that you agree that manuscript has been enhanced and that it now meets PLOS ONE’s publication criteria.

We have responded to each of the editorial and reviewers’ suggestions in numerical order below. Line numbers referenced are based on the “Revised Manuscript with Track Changes” version of the document.

**Academic Editor/Journal Requirements:**

*1. PLOS ONE style requirements*

We have reviewed the document and made some minor adjustments in line with the recommended templates. All submitted files have been named as recommended by PLOS ONE.

*2. Acknowledgements*

We have removed any reference to funding in the text. Therefore, there is no longer a need for a separate acknowledgements section, and this has now been deleted (see lines 640-643).

*3. Data Availability Statement*

The URL which links directly to the RNA-sequencing data submitted to the National Centre for Biotechnology Information (NCBI) sequence read archive has been added to the Data Availability Statement as requested.

*4. Abstract*

The discrepancy between the two abstracts was the result of the online submission form being unable to recognise Greek lettering. We have now removed these from both abstracts, and they should now be identical.

*5. Supporting Information file captions.*

We had omitted the captions for the Tables included as Supporting Information and these have now been added (see lines 802-810).

*6. Reference list*

The reference list is complete and correct. No changes have been made from the original submission.

**Reviewer # 1**

The first sentence refers specifically to the *∆tlo* mutant, while the second sentence was originally describing the *ERG*-overexpressing strains. However, to remove any ambiguity we have deleted the second sentence (*see lines 621-625*), as this is not essential.

**Reviewer # 2**

*1. Resistance phenotype*

Deletion of the *TLO* gene family results in an increase in tolerance and resistance of *C. albicans* to fluconazole. We felt that we had highlighted this in the original text, but we have reiterated the increase in resistance (*see lines 252-253*) to ensure this is now clearer.

*2. Comparison of the TLO clades*

We have included additional text (*see lines 102-105*) in the Introduction highlighting differences that we have previously observed between the gamma-clade genes and the apha- and beta-genes. In a previous study (reference [22]) we compared *TLOy11* and another gamma gene, *TLOy5*, and both were found to have identical effects, suggesting that the choice of *TLOy11* as a gamma-clade representative is sound. We have also added a sentence at the end of the Discussion (*see lines 634-638*) stating that future experiments will include additional members of each of the alpha- and gamma-*TLO* clades to see if there is intra-clade variability in Tlo protein functionality. As there is only one member of the beta clade there is no need for any further analysis of this clade.

*3. ERG251*

We agree with the reviewer (and with Reviewer #1), that the tolerance phenotype is almost certainly pleiotropic and that additional proteins are likely to be implicated. We had originally included this in the text, but have specifically referenced ERG251 in this context (*see line 597-598*) to emphasise this point.

*4. Figure 6*

The data described in Fig 6C are based on the analysis of a dataset from a previously published study (i.e. reference [22]), in which similar RNA-seq experiments were conducted on strains grown in liquid YPD medium. This has now been explicitly highlighted in the text (*see lines 432-434*)

*5. Genome assemblies.*

We have corrected the text (*see line 165*) to refer to the CGD *C. albicans* Genome Assembly 22, not Assembly 21, as originally stated.

*6. Genotypes*

S1 Table, the supplementary table detailing the genotypes of strains used in the study, has been amended, to include the addition of the *med3* deletion mutant and selection markers. Gene and strain names are now used consistently throughout the text and figures as suggested.

*7. Figures*

Fig 1A and 1C have been amended, however, S2 Fig: ergosta-5,7-dienol is already in bold

*8. Typos and syntax*

All suggested corrections have been made, although we believe that “an MIC” is correct.

We would like to reiterate our gratitude to the editors and reviewers for their very helpful comments which have certainly enhanced the manuscript. We hope that it is now suitable for publication.

With thanks and best wishes,

**Derek Sullivan	Gary Moran**

Professor in Microbiology	Associate Professor in Microbiology

School of Dental Science and	School of Dental Science and

Dublin Dental University Hospital,	Dublin Dental University Hospital,

Trinity College Dublin,	Trinity College Dublin,

Ireland	Ireland
